# Crystal structure of *N*-[(morpholin-4-yl)(thio­phen-2-yl)meth­yl]benzamide

**DOI:** 10.1107/S2056989015011639

**Published:** 2015-06-20

**Authors:** S. Arun Prabhu, M. Suresh, A. Abdul Jameel, M. Syed Ali Padusha, B. Gunasekaran

**Affiliations:** a Department of Chemistry, National College, Tiruchirappalli 620 001, Tamil Nadu, India; b PG and Research Dept of Chemistry, Jamal Mohamed College (Autonomous), Tiruchirappalli, Tamil Nadu 620 001, India; c Sri Arumugam Group of Institutions, Tholudhur, Tamil Nadu, India; d Department of Physics & Nano Technology, SRM University, SRM Nagar, Kattankulathur, Kancheepuram Dist, Chennai 603 203 Tamil Nadu, India

**Keywords:** crystal structure, benzamide, morpholino, thio­phene, hydrogen bonding

## Abstract

In the title compound, C_16_H_18_N_2_O_2_S, the morpholine ring adopts a chair conformation. The thio­phene ring makes a dihedral angle of 63.54 (14)° with the mean plane of the four C atoms [maximum deviation = 0.010 (3) Å] of the morpholine ring. The benzamide ring is disordered, with four C atoms occupying two sets of sites, with a refined occupancy ratio of 0.502 (4):0.498 (4). These two rings are inclined to one another by 85.2 (4)° and to the thio­phene ring by 72.7 (3) and 13.0 (3)° for the major and minor components, respectively. In the crystal, mol­ecules are linked *via* N—H⋯O hydrogen bonds, forming chains along [001].

## Related literature   

For the biological activity of benzamide derivatives, see: Carbonnelle *et al.* (2005 ▸); Hatzelmann & Schudt (2001 ▸); Simonini *et al.* (2006 ▸); Suzuki *et al.* (2005 ▸); Zhou *et al.* (1999 ▸); For related structures see: Muruganandam *et al.* (2009 ▸); Khan *et al.* (2012 ▸).
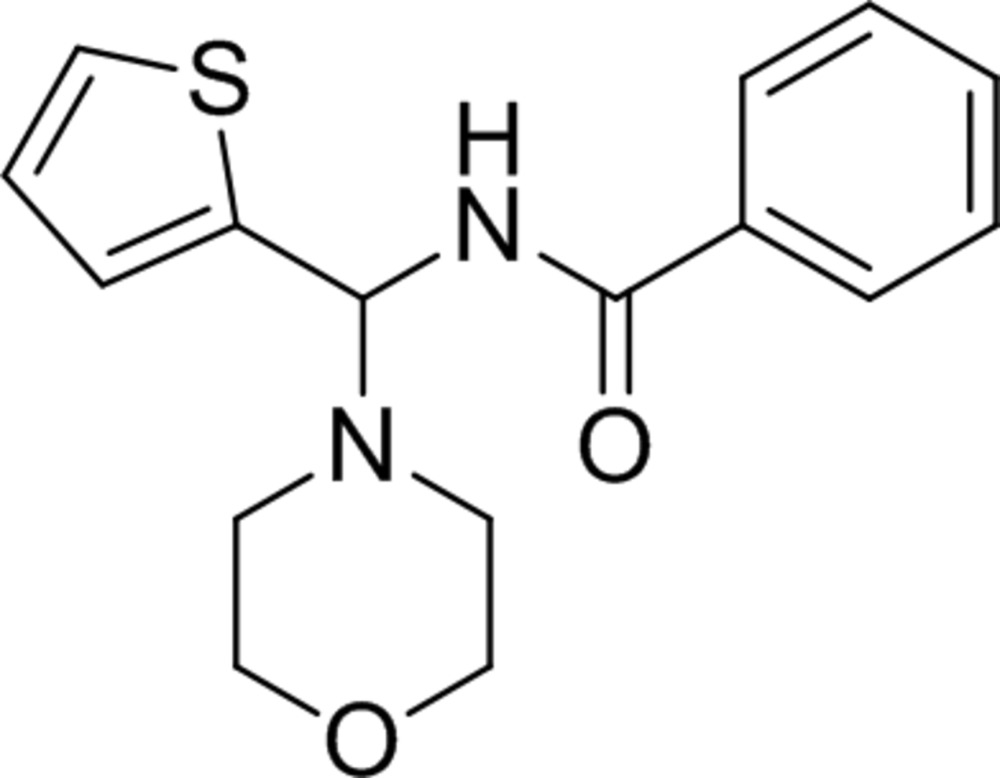


## Experimental   

### Crystal data  

C_16_H_18_N_2_O_2_S
*M* *_r_* = 302.38
Monoclinic, 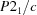

*a* = 16.5283 (11) Å
*b* = 9.9049 (7) Å
*c* = 9.6831 (5) Å
β = 99.056 (2)°
*V* = 1565.47 (17) Å^3^
*Z* = 4
Mo *K*α radiation
μ = 0.21 mm^−1^
*T* = 295 K
0.40 × 0.30 × 0.20 mm

### Data collection  

Bruker APEXII CCD diffractometer
Absorption correction: multi-scan (*SADABS*; Sheldrick, 1996 ▸) *T*_min_ = 0.920, *T*_max_ = 0.959
11905 measured reflections
3836 independent reflections
2744 reflections with *I* > 2σ(*I*)
*R*_int_ = 0.024

### Refinement  

*R*[*F* ^2^ > 2σ(*F* ^2^)] = 0.054
*wR*(*F* ^2^) = 0.182
*S* = 1.04
3836 reflections
227 parameters
1 restraint
H-atom parameters constrained
Δρ_max_ = 0.62 e Å^−3^
Δρ_min_ = −0.41 e Å^−3^

### 

Data collection: *APEX2* (Bruker, 2008 ▸); cell refinement: *SAINT* (Bruker, 2008 ▸); data reduction: *SAINT*; program(s) used to solve structure: *SHELXS97* (Sheldrick, 2008 ▸); program(s) used to refine structure: *SHELXL97* (Sheldrick, 2008 ▸); molecular graphics: *PLATON* (Spek, 2009 ▸); software used to prepare material for publication: *SHELXL97* and *PLATON*.

## Supplementary Material

Crystal structure: contains datablock(s) I. DOI: 10.1107/S2056989015011639/su5156sup1.cif

Structure factors: contains datablock(s) I. DOI: 10.1107/S2056989015011639/su5156Isup2.hkl

Supporting information file. DOI: 10.1107/S2056989015011639/su5156Isup3.cml

**Fig. 1.**
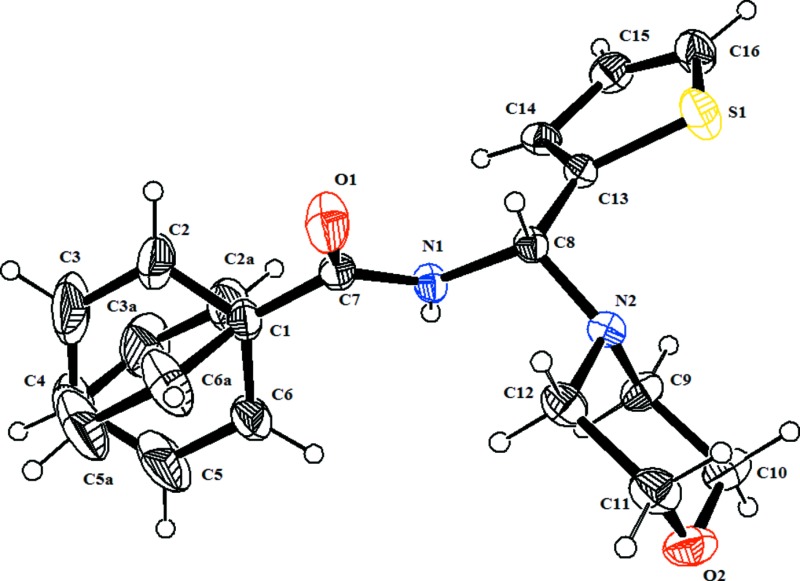
The molecular structure of the title compound, with atom labelling. Displacement ellipsoids are drawn at the 30% probability level. The minor component of the disordered benzamide ring is shown with dashed lines.

**Fig. 2.**
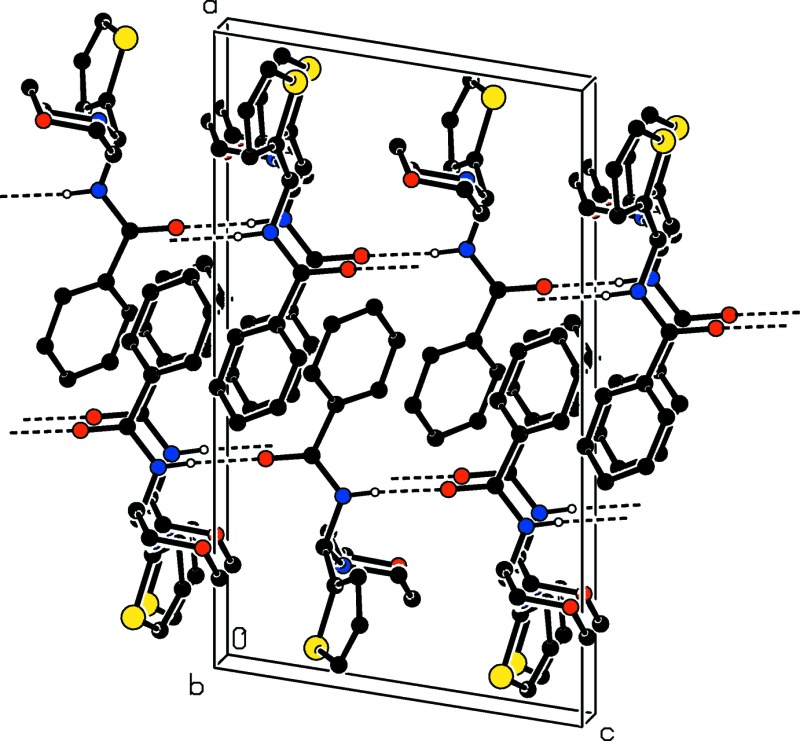
A view along the b axis of the crystal packing of the title compound. Hydrogen bonds are shown as dashed lines (see Table 1 for details). The C-bound H atoms and the minor component of the disordered benzamide ring have been omitted for clarity.

. DOI: 10.1107/S2056989015011639/su5156fig1.tif
The mol­ecular structure of the title compound, with atom labelling. Displacement ellipsoids are drawn at the 30% probability level. The minor component of the disordered benzamide ring is shown with dashed lines.

b . DOI: 10.1107/S2056989015011639/su5156fig2.tif
A view along the *b* axis of the crystal packing of the title compound. Hydrogen bonds are shown as dashed lines (see Table 1 for details). The C-bound H atoms and the minor component of the disordered benzamide ring have been omitted for clarity.

CCDC reference: 1406913

Additional supporting information:  crystallographic information; 3D view; checkCIF report

## Figures and Tables

**Table 1:** Hydrogen-bond geometry (, )

*D*H*A*	*D*H	H*A*	*D* *A*	*D*H*A*
N1H1O1^i^	0.86	2.02	2.878(2)	173

Symmetry code: (i) 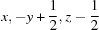.

## supplementary crystallographic information

### S1. Synthesis and crystallization

To an alkaline solution of benzamide (0.025 mol, 3.03 g), morpholine (0.025 mol, 2.2 ml) was added drop wise in ice cold conditions and the contents were stirred for 5 min. Thio­phene-2-aldehyde (0.025 mol, 2.3 ml) was then added drop wise and stirring was continued for 1 h. The Mannich base product formed was filtered, washed with water and recrystallized with ethanol (yield: 75%; m.p.: 433 K) giving colourless block-like crystals.

### S2. Refinement

Crystal data, data collection and structure refinement details are summarized in Table 2. Four C atoms in the benzamide ring (C2/C2A, C3/C3A, C5/C5A, and C6/C6A) are disordered over two positions with a refined occupany ratio of 0.502 (5):0.48 (5). All of the H atoms were positioned geometrically and refined using a riding model: N—H = 0.86 Å, C—H = 0.93 - 0.98 Å with *U*_iso_(H) = 1.2U_eq_(N,C).

### S3. Comment

Benzamide and its derivatives, have recently received great attention because of their wide range of pharmacological activities, such as anti-inflammatory, immunomodulatory (Hatzelmann & Schudt, 2001; Carbonnelle *et al.*, 2005), anti-tumoral (Suzuki *et al.*, 2005), anti­psychotic (Simonini *et al.*, 2006), and anti­allergic (Zhou *et al.*, 1999).

The geometric parameters of the title compound (Fig. 1) agree well with those reported for similar structures (Muruganandam *et al.*, 2009; Khan *et al.*, 2012). The morpholine ring adopts a chair conformation. The thio­phene ring makes a dihedral angle of 63.54 (14) ° with the mean plane of the four C atoms (maximum deviation 0.010 (3) Å) of the morpholine ring. The benzamide ring is disordered with four C atoms occupying two positions, with a refined occupancy ratio of 0.502 (5):0.498 (5). These two rings are inclined to one another by 85.2 (4) ° and to the thio­phene ring by 72.7 (3) and 13.0 (3) °, for the major and minor component, respectively.

In the crystal, molecules are linked via N—H···O hydrogen bonds forming chains along [001]; see Table 1 and Fig. 2

### Crystal data

**Table:**

C_16_H_18_N_2_O_2_S	*F*(000) = 640
*M**_r_* = 302.38	*D*_x_ = 1.283 Mg m^−^^3^
Monoclinic, *P*2_1_/*c*	Mo *K*α radiation, λ = 0.71073 Å
Hall symbol: -P 2ybc	Cell parameters from 3836 reflections
*a* = 16.5283 (11) Å	θ = 2.4–28.3°
*b* = 9.9049 (7) Å	µ = 0.21 mm^−^^1^
*c* = 9.6831 (5) Å	*T* = 295 K
β = 99.056 (2)°	Block, colourless
*V* = 1565.47 (17) Å^3^	0.40 × 0.30 × 0.20 mm
*Z* = 4	

### Data collection

**Table:**

Bruker APEXII CCD diffractometer	3836 independent reflections
Radiation source: fine-focus sealed tube	2744 reflections with *I* > 2σ(*I*)
Graphite monochromator	*R*_int_ = 0.024
Detector resolution: 0 pixels mm^-1^	θ_max_ = 28.3°, θ_min_ = 2.4°
ω and φ scans	*h* = −21→21
Absorption correction: multi-scan (*SADABS*; Sheldrick, 1996)	*k* = −13→13
*T*_min_ = 0.920, *T*_max_ = 0.959	*l* = −12→7
11905 measured reflections	

### Refinement

**Table:**

Refinement on *F*^2^	Primary atom site location: structure-invariant direct methods
Least-squares matrix: full	Secondary atom site location: difference Fourier map
*R*[*F*^2^ > 2σ(*F*^2^)] = 0.054	Hydrogen site location: inferred from neighbouring sites
*wR*(*F*^2^) = 0.182	H-atom parameters constrained
*S* = 1.04	*w* = 1/[σ^2^(*F*_o_^2^) + (0.097*P*)^2^ + 0.5504*P*] where *P* = (*F*_o_^2^ + 2*F*_c_^2^)/3
3836 reflections	(Δ/σ)_max_ < 0.001
227 parameters	Δρ_max_ = 0.62 e Å^−^^3^
1 restraint	Δρ_min_ = −0.41 e Å^−^^3^

### Fractional atomic coordinates and isotropic or equivalent isotropic displacement parameters (Å^2^)

**Table:**

	*x*	*y*	*z*	*U*_iso_*/*U*_eq_	Occ. (<1)
S1	0.94818 (4)	0.28631 (7)	0.23066 (8)	0.0622 (3)	
N2	0.81748 (10)	0.05848 (16)	0.16552 (16)	0.0345 (4)	
O2	0.81001 (12)	−0.19318 (16)	0.0245 (2)	0.0610 (5)	
O1	0.66536 (11)	0.2032 (3)	0.37090 (16)	0.0734 (7)	
N1	0.70424 (10)	0.21935 (18)	0.16023 (16)	0.0365 (4)	
H1	0.6897	0.2365	0.0728	0.044*	
C14	0.82300 (13)	0.4243 (2)	0.1121 (2)	0.0406 (5)	
H14	0.7698	0.4523	0.0784	0.049*	
C7	0.64812 (12)	0.2222 (2)	0.2448 (2)	0.0402 (5)	
C1	0.56136 (13)	0.2450 (2)	0.1788 (2)	0.0450 (5)	
C2	0.5166 (4)	0.3348 (8)	0.2408 (8)	0.087 (2)	0.502 (4)
H2	0.5404	0.3854	0.3174	0.105*	0.502 (4)
C6	0.5297 (3)	0.1744 (7)	0.0642 (6)	0.0662 (16)	0.502 (4)
H6	0.5626	0.1187	0.0192	0.079*	0.502 (4)
C2A	0.5393 (3)	0.3477 (7)	0.0839 (7)	0.0745 (18)	0.498 (4)
H2A	0.5798	0.4024	0.0570	0.089*	0.498 (4)
C6A	0.4971 (4)	0.1642 (9)	0.2132 (8)	0.093 (3)	0.498 (4)
H6A	0.5096	0.0944	0.2772	0.112*	0.498 (4)
C15	0.89612 (15)	0.5008 (2)	0.1046 (3)	0.0502 (5)	
H15	0.8954	0.5852	0.0624	0.060*	
C13	0.84490 (11)	0.29981 (19)	0.1788 (2)	0.0342 (4)	
C8	0.79002 (11)	0.1877 (2)	0.21266 (19)	0.0329 (4)	
H8	0.7955	0.1831	0.3148	0.039*	
C16	0.96545 (15)	0.4401 (2)	0.1637 (3)	0.0570 (6)	
H16	1.0172	0.4781	0.1676	0.068*	
C9	0.81880 (14)	0.0498 (2)	0.0153 (2)	0.0432 (5)	
H9A	0.8509	0.1235	−0.0138	0.052*	
H9B	0.7635	0.0569	−0.0355	0.052*	
C10	0.85569 (17)	−0.0830 (2)	−0.0167 (3)	0.0549 (6)	
H10A	0.8572	−0.0888	−0.1163	0.066*	
H10B	0.9116	−0.0881	0.0319	0.066*	
C12	0.77106 (14)	−0.0547 (2)	0.2094 (2)	0.0463 (5)	
H12A	0.7143	−0.0484	0.1649	0.056*	
H12B	0.7725	−0.0516	0.3098	0.056*	
C11	0.80761 (16)	−0.1865 (2)	0.1691 (3)	0.0557 (6)	
H11A	0.8628	−0.1956	0.2200	0.067*	
H11B	0.7753	−0.2612	0.1955	0.067*	
C4	0.3980 (2)	0.2798 (6)	0.0732 (5)	0.1096 (16)	
H4	0.3428	0.2933	0.0388	0.132*	
C3	0.4339 (4)	0.3488 (11)	0.1858 (11)	0.114 (3)	0.502 (4)
H3	0.4023	0.4084	0.2289	0.137*	0.502 (4)
C5	0.4464 (4)	0.1872 (11)	0.0152 (8)	0.106 (3)	0.502 (4)
H5	0.4224	0.1326	−0.0580	0.127*	0.502 (4)
C3A	0.4572 (4)	0.3710 (10)	0.0275 (8)	0.101 (3)	0.498 (4)
H3A	0.4416	0.4407	−0.0354	0.121*	0.498 (4)
C5A	0.4167 (4)	0.1852 (13)	0.1551 (11)	0.114 (3)	0.498 (4)
H5A	0.3762	0.1279	0.1777	0.136*	0.498 (4)

### Atomic displacement parameters (Å^2^)

**Table:**

	*U*^11^	*U*^22^	*U*^33^	*U*^12^	*U*^13^	*U*^23^
S1	0.0364 (3)	0.0508 (4)	0.0969 (6)	−0.0011 (2)	0.0025 (3)	0.0151 (3)
N2	0.0359 (8)	0.0349 (8)	0.0319 (8)	−0.0025 (6)	0.0033 (6)	0.0022 (6)
O2	0.0749 (12)	0.0380 (9)	0.0652 (11)	0.0012 (8)	−0.0042 (9)	−0.0067 (7)
O1	0.0502 (10)	0.142 (2)	0.0284 (8)	0.0135 (11)	0.0070 (7)	−0.0040 (9)
N1	0.0306 (8)	0.0520 (10)	0.0258 (7)	0.0022 (7)	0.0014 (6)	0.0014 (7)
C14	0.0478 (10)	0.0426 (11)	0.0307 (9)	−0.0071 (8)	0.0041 (8)	−0.0024 (8)
C7	0.0352 (10)	0.0549 (12)	0.0300 (9)	−0.0002 (8)	0.0039 (7)	−0.0081 (8)
C1	0.0327 (10)	0.0642 (14)	0.0385 (10)	0.0004 (9)	0.0069 (8)	−0.0069 (10)
C2	0.046 (3)	0.120 (6)	0.096 (5)	0.021 (3)	0.013 (3)	−0.035 (4)
C6	0.038 (2)	0.097 (4)	0.062 (3)	−0.006 (2)	0.000 (2)	−0.020 (3)
C2A	0.050 (3)	0.085 (4)	0.086 (4)	0.016 (3)	0.004 (3)	0.003 (3)
C6A	0.046 (3)	0.141 (7)	0.091 (5)	−0.020 (4)	0.006 (3)	0.019 (5)
C15	0.0563 (12)	0.0358 (11)	0.0592 (13)	−0.0036 (9)	0.0115 (11)	0.0041 (10)
C13	0.0313 (9)	0.0365 (10)	0.0339 (9)	0.0006 (7)	0.0029 (7)	−0.0036 (7)
C8	0.0297 (8)	0.0420 (10)	0.0261 (8)	0.0008 (7)	0.0019 (7)	0.0001 (7)
C16	0.0446 (12)	0.0454 (13)	0.0821 (18)	−0.0088 (10)	0.0136 (11)	0.0029 (12)
C9	0.0565 (12)	0.0396 (11)	0.0332 (10)	0.0059 (9)	0.0061 (9)	0.0000 (8)
C10	0.0717 (16)	0.0452 (13)	0.0471 (12)	0.0089 (11)	0.0072 (11)	−0.0057 (10)
C12	0.0441 (11)	0.0448 (12)	0.0488 (12)	−0.0082 (9)	0.0039 (9)	0.0100 (9)
C11	0.0565 (14)	0.0397 (12)	0.0677 (16)	−0.0052 (10)	−0.0004 (12)	0.0114 (11)
C4	0.0342 (15)	0.188 (5)	0.102 (3)	0.011 (2)	−0.0034 (17)	−0.028 (3)
C3	0.050 (4)	0.155 (9)	0.141 (8)	0.040 (5)	0.027 (4)	−0.010 (6)
C5	0.048 (3)	0.179 (9)	0.082 (5)	−0.027 (4)	−0.018 (3)	−0.008 (5)
C3A	0.072 (4)	0.129 (7)	0.094 (5)	0.047 (5)	−0.010 (4)	0.002 (5)
C5A	0.039 (3)	0.176 (10)	0.122 (7)	−0.020 (5)	0.000 (4)	0.008 (7)

### Geometric parameters (Å, º)

**Table:**

S1—C16	1.697 (2)	C6A—H6A	0.9300
S1—C13	1.7071 (19)	C15—C16	1.340 (3)
N2—C8	1.455 (2)	C15—H15	0.9300
N2—C12	1.459 (2)	C13—C8	1.502 (3)
N2—C9	1.461 (2)	C8—H8	0.9800
O2—C11	1.408 (3)	C16—H16	0.9300
O2—C10	1.420 (3)	C9—C10	1.503 (3)
O1—C7	1.223 (3)	C9—H9A	0.9700
N1—C7	1.331 (3)	C9—H9B	0.9700
N1—C8	1.463 (2)	C10—H10A	0.9700
N1—H1	0.8600	C10—H10B	0.9700
C14—C13	1.413 (3)	C12—C11	1.516 (3)
C14—C15	1.438 (3)	C12—H12A	0.9700
C14—H14	0.9300	C12—H12B	0.9700
C7—C1	1.493 (3)	C11—H11A	0.9700
C1—C6	1.345 (6)	C11—H11B	0.9700
C1—C2	1.356 (6)	C4—C5A	1.235 (11)
C1—C2A	1.380 (7)	C4—C3	1.344 (11)
C1—C6A	1.411 (7)	C4—C5	1.392 (10)
C2—C3	1.394 (9)	C4—C3A	1.451 (11)
C2—H2	0.9300	C4—H4	0.9300
C6—C5	1.391 (7)	C3—H3	0.9300
C6—H6	0.9300	C5—H5	0.9300
C2A—C3A	1.400 (8)	C3A—H3A	0.9300
C2A—H2A	0.9300	C5A—H5A	0.9300
C6A—C5A	1.375 (10)		
			
C16—S1—C13	92.16 (11)	C15—C16—H16	123.8
C8—N2—C12	112.35 (15)	S1—C16—H16	123.8
C8—N2—C9	114.73 (15)	N2—C9—C10	109.09 (17)
C12—N2—C9	109.61 (16)	N2—C9—H9A	109.9
C11—O2—C10	110.06 (18)	C10—C9—H9A	109.9
C7—N1—C8	121.48 (15)	N2—C9—H9B	109.9
C7—N1—H1	119.3	C10—C9—H9B	109.9
C8—N1—H1	119.3	H9A—C9—H9B	108.3
C13—C14—C15	109.02 (19)	O2—C10—C9	111.3 (2)
C13—C14—H14	125.5	O2—C10—H10A	109.4
C15—C14—H14	125.5	C9—C10—H10A	109.4
O1—C7—N1	122.37 (19)	O2—C10—H10B	109.4
O1—C7—C1	120.54 (19)	C9—C10—H10B	109.4
N1—C7—C1	117.06 (17)	H10A—C10—H10B	108.0
C6—C1—C2	122.5 (4)	N2—C12—C11	109.75 (18)
C6—C1—C2A	78.9 (4)	N2—C12—H12A	109.7
C2—C1—C2A	72.9 (5)	C11—C12—H12A	109.7
C6—C1—C6A	72.0 (4)	N2—C12—H12B	109.7
C2—C1—C6A	77.6 (5)	C11—C12—H12B	109.7
C2A—C1—C6A	116.5 (4)	H12A—C12—H12B	108.2
C6—C1—C7	119.8 (3)	O2—C11—C12	111.78 (19)
C2—C1—C7	117.6 (3)	O2—C11—H11A	109.3
C2A—C1—C7	122.2 (3)	C12—C11—H11A	109.3
C6A—C1—C7	121.4 (4)	O2—C11—H11B	109.3
C1—C2—C3	117.8 (7)	C12—C11—H11B	109.3
C1—C2—H2	121.1	H11A—C11—H11B	107.9
C3—C2—H2	121.1	C5A—C4—C3	80.2 (7)
C1—C6—C5	118.0 (6)	C5A—C4—C5	70.0 (7)
C1—C6—H6	121.0	C3—C4—C5	117.0 (5)
C5—C6—H6	121.0	C5A—C4—C3A	123.7 (5)
C1—C2A—C3A	121.3 (6)	C3—C4—C3A	72.4 (6)
C1—C2A—H2A	119.4	C5—C4—C3A	80.2 (5)
C3A—C2A—H2A	119.4	C5A—C4—H4	118.1
C5A—C6A—C1	122.2 (7)	C3—C4—H4	120.1
C5A—C6A—H6A	118.9	C5—C4—H4	123.0
C1—C6A—H6A	118.9	C3A—C4—H4	118.1
C16—C15—C14	114.3 (2)	C4—C3—C2	122.8 (7)
C16—C15—H15	122.8	C4—C3—H3	118.6
C14—C15—H15	122.8	C2—C3—H3	118.6
C14—C13—C8	128.68 (17)	C6—C5—C4	121.6 (6)
C14—C13—S1	112.05 (15)	C6—C5—H5	119.2
C8—C13—S1	119.20 (14)	C4—C5—H5	119.2
N2—C8—N1	114.37 (15)	C2A—C3A—C4	115.9 (7)
N2—C8—C13	110.65 (14)	C2A—C3A—H3A	122.0
N1—C8—C13	110.57 (16)	C4—C3A—H3A	122.0
N2—C8—H8	106.9	C4—C5A—C6A	120.4 (8)
N1—C8—H8	106.9	C4—C5A—H5A	119.8
C13—C8—H8	106.9	C6A—C5A—H5A	119.8
C15—C16—S1	112.44 (18)		
			
C8—N1—C7—O1	2.6 (3)	C9—N2—C8—C13	−59.9 (2)
C8—N1—C7—C1	−175.38 (18)	C7—N1—C8—N2	111.0 (2)
O1—C7—C1—C6	−130.9 (4)	C7—N1—C8—C13	−123.3 (2)
N1—C7—C1—C6	47.1 (4)	C14—C13—C8—N2	130.5 (2)
O1—C7—C1—C2	47.1 (5)	S1—C13—C8—N2	−52.7 (2)
N1—C7—C1—C2	−134.9 (5)	C14—C13—C8—N1	2.7 (3)
O1—C7—C1—C2A	133.5 (4)	S1—C13—C8—N1	179.57 (13)
N1—C7—C1—C2A	−48.5 (4)	C14—C15—C16—S1	0.7 (3)
O1—C7—C1—C6A	−44.8 (5)	C13—S1—C16—C15	0.3 (2)
N1—C7—C1—C6A	133.1 (5)	C8—N2—C9—C10	174.63 (17)
C6—C1—C2—C3	2.8 (10)	C12—N2—C9—C10	−57.9 (2)
C2A—C1—C2—C3	67.0 (8)	C11—O2—C10—C9	−59.3 (3)
C6A—C1—C2—C3	−56.0 (8)	N2—C9—C10—O2	59.6 (3)
C7—C1—C2—C3	−175.2 (6)	C8—N2—C12—C11	−174.71 (17)
C2—C1—C6—C5	−5.1 (9)	C9—N2—C12—C11	56.5 (2)
C2A—C1—C6—C5	−66.4 (7)	C10—O2—C11—C12	57.7 (3)
C6A—C1—C6—C5	56.4 (7)	N2—C12—C11—O2	−56.9 (2)
C7—C1—C6—C5	172.8 (5)	C5A—C4—C3—C2	64.2 (10)
C6—C1—C2A—C3A	64.4 (7)	C5—C4—C3—C2	2.7 (14)
C2—C1—C2A—C3A	−65.0 (7)	C3A—C4—C3—C2	−66.0 (10)
C6A—C1—C2A—C3A	1.1 (9)	C1—C2—C3—C4	−1.6 (14)
C7—C1—C2A—C3A	−177.3 (5)	C1—C6—C5—C4	6.3 (11)
C6—C1—C6A—C5A	−66.7 (8)	C5A—C4—C5—C6	−72.3 (9)
C2—C1—C6A—C5A	63.9 (9)	C3—C4—C5—C6	−5.1 (12)
C2A—C1—C6A—C5A	0.4 (10)	C3A—C4—C5—C6	59.2 (8)
C7—C1—C6A—C5A	178.9 (7)	C1—C2A—C3A—C4	−1.0 (10)
C13—C14—C15—C16	−1.6 (3)	C5A—C4—C3A—C2A	−1.0 (11)
C15—C14—C13—C8	178.82 (19)	C3—C4—C3A—C2A	63.8 (7)
C15—C14—C13—S1	1.8 (2)	C5—C4—C3A—C2A	−58.7 (7)
C16—S1—C13—C14	−1.24 (17)	C3—C4—C5A—C6A	−58.5 (9)
C16—S1—C13—C8	−178.59 (17)	C5—C4—C5A—C6A	65.1 (9)
C12—N2—C8—N1	−60.4 (2)	C3A—C4—C5A—C6A	2.6 (14)
C9—N2—C8—N1	65.7 (2)	C1—C6A—C5A—C4	−2.3 (14)
C12—N2—C8—C13	173.99 (15)		

### Hydrogen-bond geometry (Å, º)

**Table:**

*D*—H···*A*	*D*—H	H···*A*	*D*···*A*	*D*—H···*A*
N1—H1···O1^i^	0.86	2.02	2.878 (2)	173

Symmetry code: (i) *x*, −*y*+1/2, *z*−1/2.
