# Supplementary figures and images for: Crystal structure of N-[(morpholin-4-yl)(thio­phen-2-yl)meth­yl]benzamide
Source: Acta Crystallogr E Crystallogr Commun. 2015 Jun 20;71(Pt 7):o498–9. doi: 10.1107/S2056989015011639 (PMC4518906; doi:10.1107/S2056989015011639)

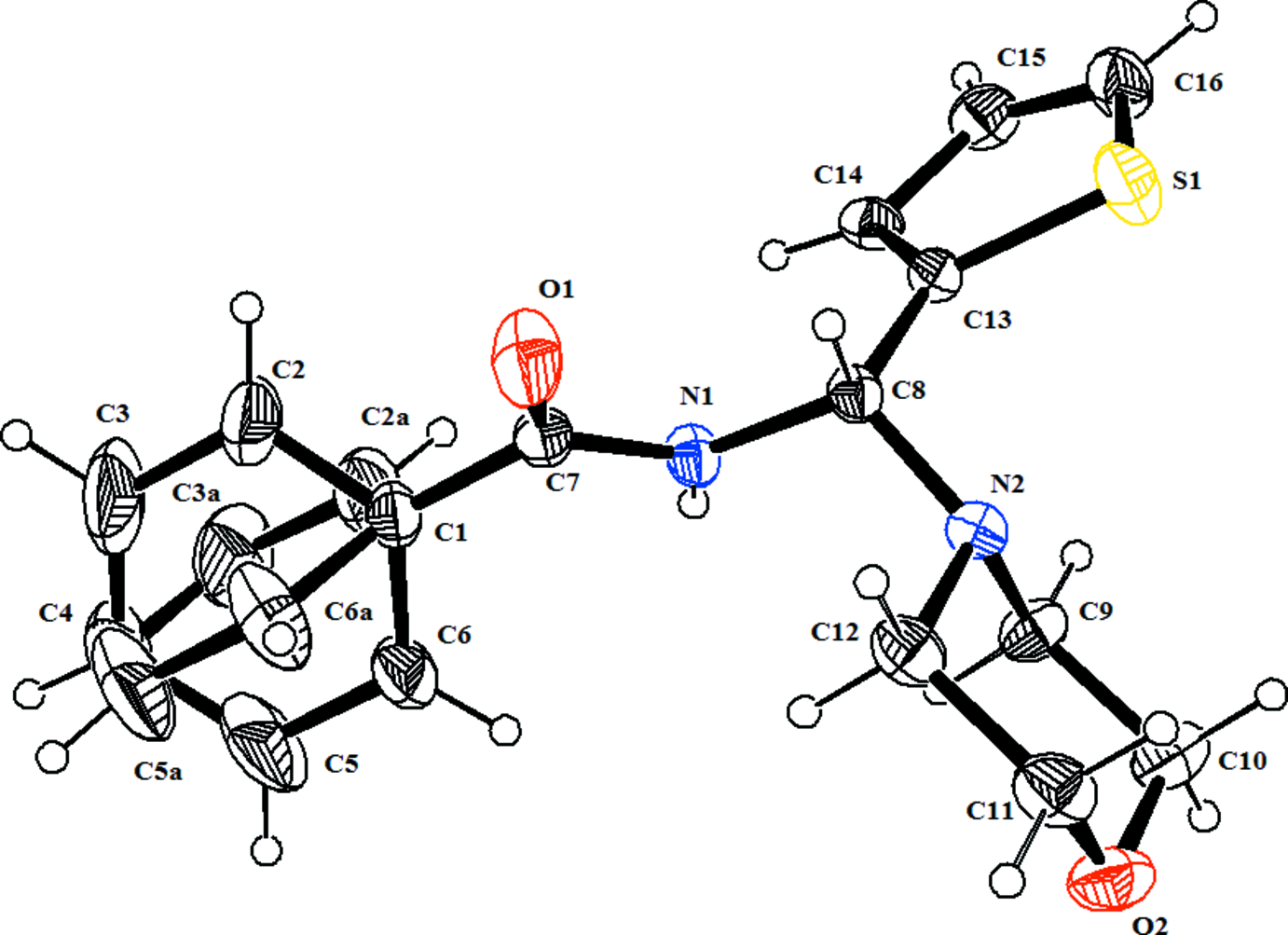

Supplement: Supplementary file 4 [file e-71-0o498-fig1.tif]

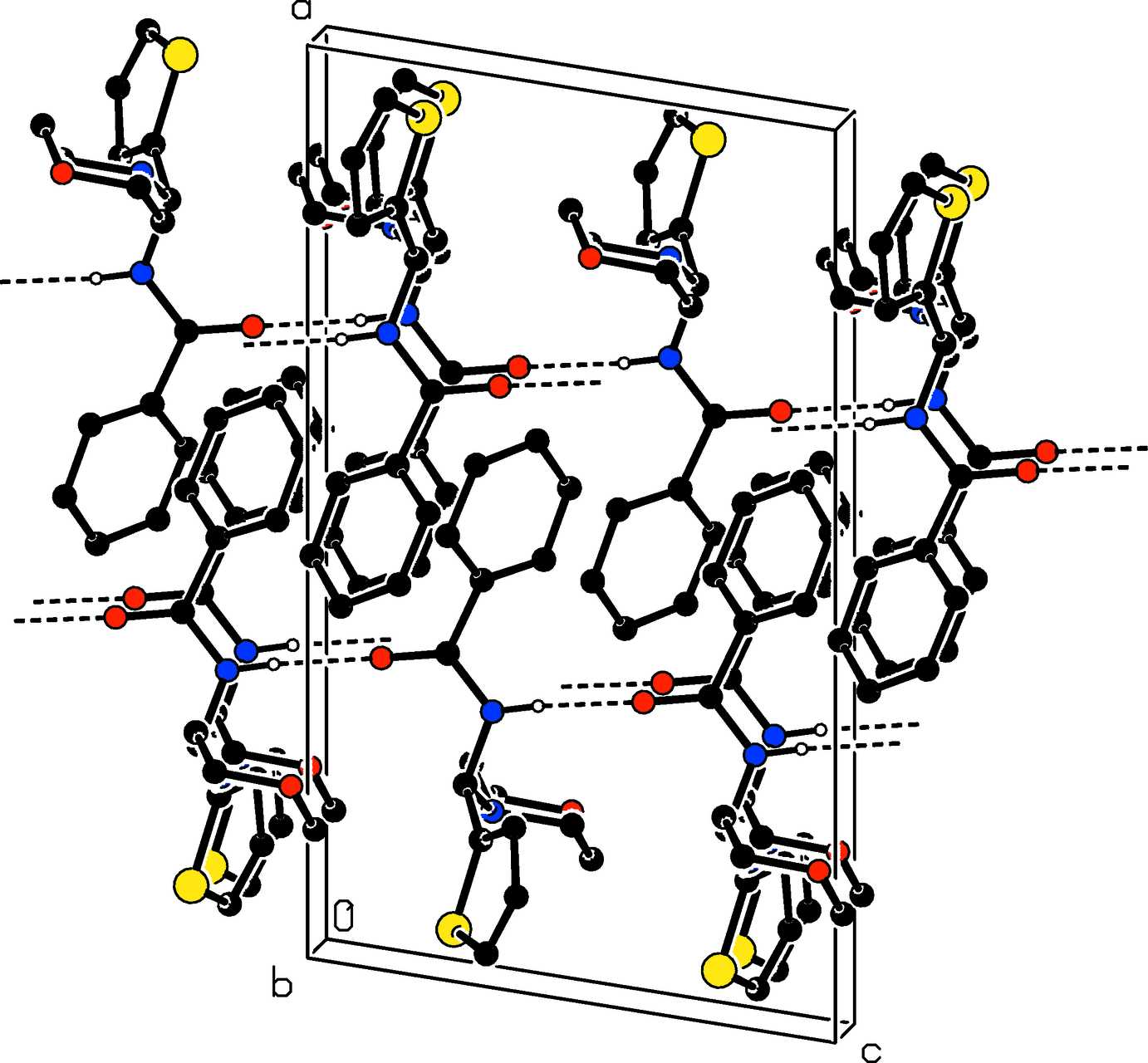

Supplement: Supplementary file 5 [file e-71-0o498-fig2.tif]
